# COVID-19 vaccine inequity in African low-income countries

**DOI:** 10.3389/fpubh.2023.1087662

**Published:** 2023-03-06

**Authors:** Chifundo Annessia Kunyenje, Gowokani Chijere Chirwa, Sebastian M. Mboma, Wingston Ng'ambi, Emmanuel Mnjowe, Dominic Nkhoma, Lucky Gift Ngwira, Marlen Stacey Chawani, Ben Chilima, Collins Mitambo, Amelia Crampin, Joseph Mfutso-Bengo

**Affiliations:** ^1^Department of Health Systems, Health Economics and Policy Unit, Kamuzu University of Health Sciences (KUHeS), Lilongwe, Malawi; ^2^Department of Economics, Chancellor College, University of Malawi, Zomba, Malawi; ^3^Malawi Liverpool Wellcome Programme, Policy Unit, Lilongwe, Malawi; ^4^Ministry of Health, Public Health Institute of Malawi, Lilongwe, Malawi; ^5^Ministry of Heath, Research Directorate, Lilongwe, Malawi; ^6^Malawi Epidemiology and Intervention Research Unit, Lilongwe, Malawi

**Keywords:** COVID-19 vaccine, vaccine equity, vaccine coverage, distribution, uptake, low-income countries

## Abstract

Equitable access and utilization of the COVID-19 vaccine is the main exit strategy from the pandemic. This paper used proceedings from the Second Extraordinary Think-Tank conference, which was held by the Health Economics and Policy Unit at the Kamuzu University of Health Sciences in collaboration with the Malawi Ministry of Health, complemented by a review of literature. We found disparities in COVID-19 vaccine coverage among low-income countries. This is also the case among high income countries. The disparities are driven mainly by insufficient supply, inequitable distribution, limited production of the vaccine in low-income countries, weak health systems, high vaccine hesitancy, and vaccine misconceptions. COVID-19 vaccine inequity continues to affect the entire world with the ongoing risks of emergence of new COVID-19 variants, increased morbidity and mortality and social and economic disruptions. In order to reduce the COVID-19 vaccination inequality in low-income countries, there is need to expand COVAX facility, waive intellectual property rights, transform knowledge and technology acquired into vaccines, and conduct mass COVID-19 vaccination campaigns.

## 1. Introduction

Coronavirus Disease 2019 (COVID-19), caused by the SARS-CoV-2, has ravaged the globe. To date, there have been more than half a billion confirmed cases and more than 6 million deaths globally (1). Early in the pandemic, many countries adhered to such measures as physical distancing; hand washing, hand sanitizers and face masks protocols; isolation/quarantine; and lockdown to reduce the spread of the COVID-19 (2). Despite reducing morbidity and mortality rates, these measures had severe impacts on economies, social life and freedom of the people in the world (3). Vaccination became the expedient eradication strategy to ensure the globe returns to normalcy (4).

Within a year after the emergence of COVID-19, there was rapid development, distribution and administration of COVID-19 vaccines across the globe, including Pfizer-BioNTech Moderna, Johnson & Johnson; the Oxford-AstraZeneca; and other brands (5). The speedy development and approval of safe and effective vaccines was only the first step to ending the pandemic, but vaccine equity- “a situation where all individuals, populations and countries have equitable access to vaccine without incurring financial hardship” (6), is necessary to overcome the pandemic and achieve enough global protection. Nevertheless, the scope of global COVID-19 vaccine inequity is enormous especially in low-income countries (LICs), many of which are in Africa, and the consequences continue to negatively impact the world (7). This paper provides an overview of the COVID-19 vaccine inequity in LICs by December 2022.

## 2. Methods

This perspective paper used proceedings from the Second Extraordinary Think-Tank conference, which was held by the Health Economics and Policy Unit at the Kamuzu University of Health Sciences in collaboration with the Malawi Ministry of Health. The main approach involved a comprehensive review of literature. Google search engine was used to search for eligible literature and search terms included the following: “COVID-19 vaccine,” “Equitable access to COVID-19 vaccine,” “Equitable distribution of COVID-19 vaccine,” “Low-income countries,” “High-income countries,” “COVID-19 vaccine coverage,” and “COVID-19 vaccine uptake.” Studies and articles found were collated and reviewed to extract content related to the topic under review.

The data visualizations on COVID-19 vaccination coverage and doses purchased, disaggregated by country income, which were used in the paper were retrieved from the online databases: https://ourworldindata.org/covid-vaccinations and Duke Global Health Innovation Center. Country income classifications were obtained from the World Bank to compare COVID-19 vaccine coverage among the top 10 richest countries to the bottom 10 poorest countries.

## 3. Results

### 3.1. The scope of COVID-19 vaccine inequity

**Figure 1:**
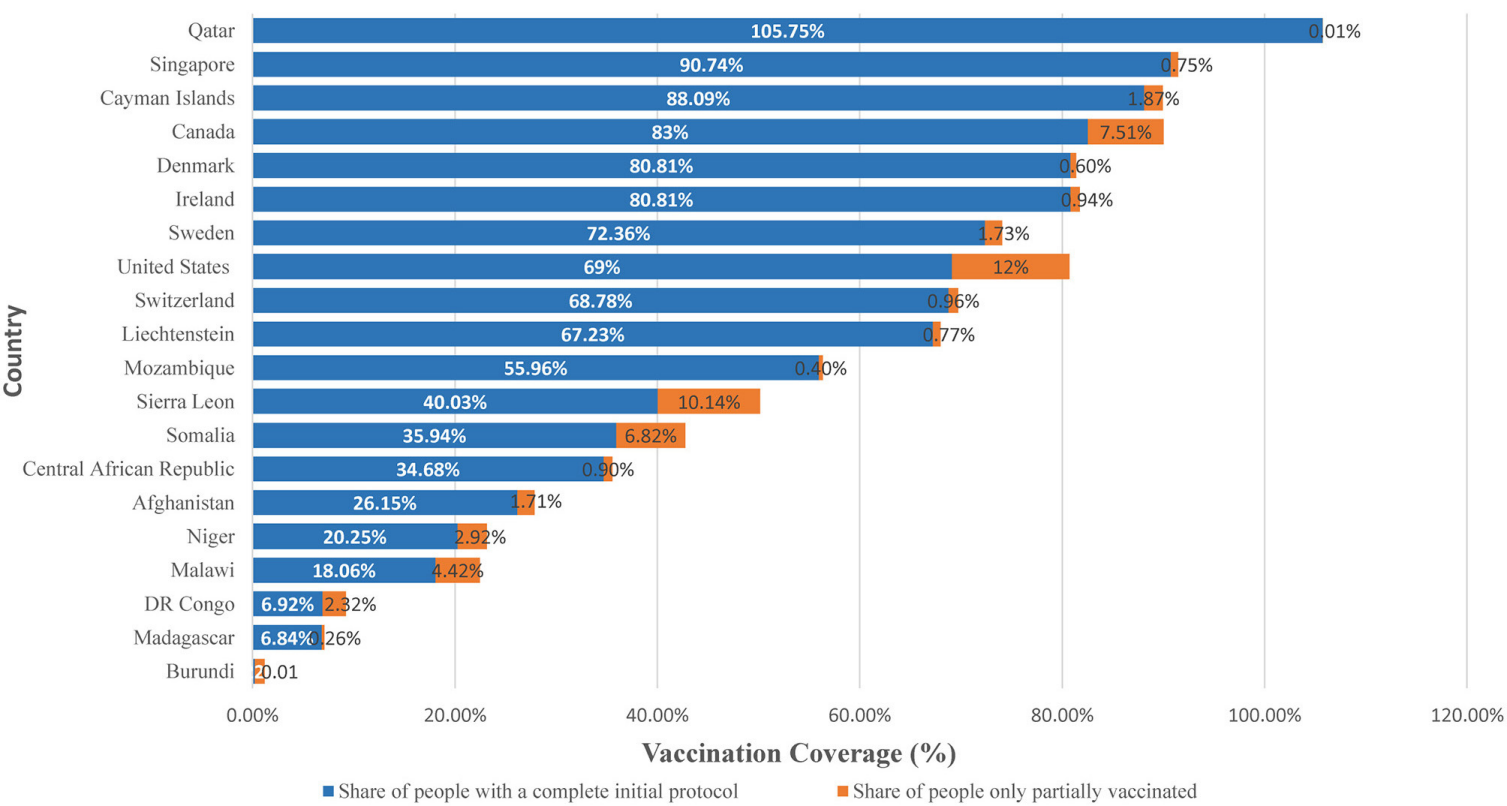
Share of people vaccinated against COVID-19 vaccine among top 10 HICs and bottom 10 LICs, December 2022. Source: Official data collated by Our World in Data (1).

Globally, there is higher coverage of COVID-19 vaccination in high income countries (HICs) than LICs (8). The disparities within LICs are greater than within the HICs. Figure 1 shows the share of people fully and partially vaccinated in the top 10 HICs and bottom 10 LICs (1). Among the top 10 HICs, COVID-19 vaccine coverage was highest in Qatar (105.75%%) and lowest in the Liechtenstein (67.23%). Among the bottom 10 LICs, COVID-19 vaccine coverage was highest in Mozambique (55.96%) and lowest in Burundi (<1%).

More than a year since the COVID-19 Vaccines Global Access (COVAX) facility initiated the delivery of the COVID-19 vaccines to LICs, distribution of the COVID-19 vaccine improved (9). Among the continents, Africa continues to experience the lowest vaccination coverage with only 28% of its population fully vaccinated, as of December 2022 (1). In Africa, 15 countries were yet to reach 10% full vaccination of their population; 21 countries had reached between 10 and 19% of their population; five countries had reached between 40 and 69% of their population; and only Mauritius, Rwanda and Seychelles surpassed 70% vaccination coverage (9). Across the globe, the COVID-19 vaccination coverage gap is more profound between the urban and rural areas. Urban areas had higher COVID-19 vaccination coverage than rural areas both in HICs and LICs. A study in the USA showed that despite the increased availability and access to COVID-19 vaccines, COVID-19 vaccination coverage with the first dose was lower in rural (58.5%) than in urban areas (75.4%) (10). The disparities almost doubled since April 2021 through January 2022, and persisted across sex and age groups (10). In Malawi, vaccines were primarily distributed in the urban areas, excluding most rural areas where the majority (80%) of the population is located (11). In rural areas of Malawi, after vaccine was widely available almost 98% of the population reported not having been offered the COVID-19 vaccine and another 1% were offered but refused to be vaccinated (12). This corroborates with Achim Steiner, UNDP Administrator, that < 1% of the population in some LICs is vaccinated (13).

### 3.2. Barriers to vaccine equity in LICs

COVID-19 vaccines ought to be equitably accessed and distributed globally to achieve enough global protection and to ensure the globe returns to normalcy. However, COVID-19 vaccine inequity remains a challenge, largely due to the following factors.

#### 3.2.1. Low funding

This leads to unaffordability and inaccessibility of COVID-19 vaccine in the LICs (14, 15). With low funding, LICs find themselves in ethical dilemmas of either: (a) completely transferring funds that were meant to finance the rest of the national health system; (b) increasing her loan burden by borrowing from the World Bank COVID-19 vaccine loan facility, AU AVATT COVID-19 vaccine mechanism or Afrexim Bank COVID-19 vaccine facility; (c) inequitably leave the rest of population behind; or (d) wait for the goodwill of COVAX COVID-19 vaccine facility to increase its allocation.

#### 3.2.2. Problem of intellectual property rights

Although vaccines are a global public good, obtaining intellectual property (IP) for COVID-19 vaccines by LICs from vaccine developers remained difficult (16). Initially, India and South Africa proposed manufacturers in HICs to relinquish intellectual property rights associated with COVID-19 vaccines and therapeutics to increase manufacturing and access in LICs; but faced strong opposition from several members of the European Union (EU) and UK (17). Nevertheless, unrestricted access to IP of COVID-19 vaccine was later granted to a few countries like India, the Republic of Korea, Brazil, Indonesia, South Africa, Egypt, Morocco, Senegal, and Tunisia, which have already started producing vaccines (5, 18). Despite this development, LICs cannot easily take advantage of this due to weak systems like lack of domestic vaccine manufacturers; and lack of vaccine-producing resources such as well-equipped laboratories, research and developments, policy, programs, and government funding.

#### 3.2.3. Vaccine nationalism

**Figure 2:**
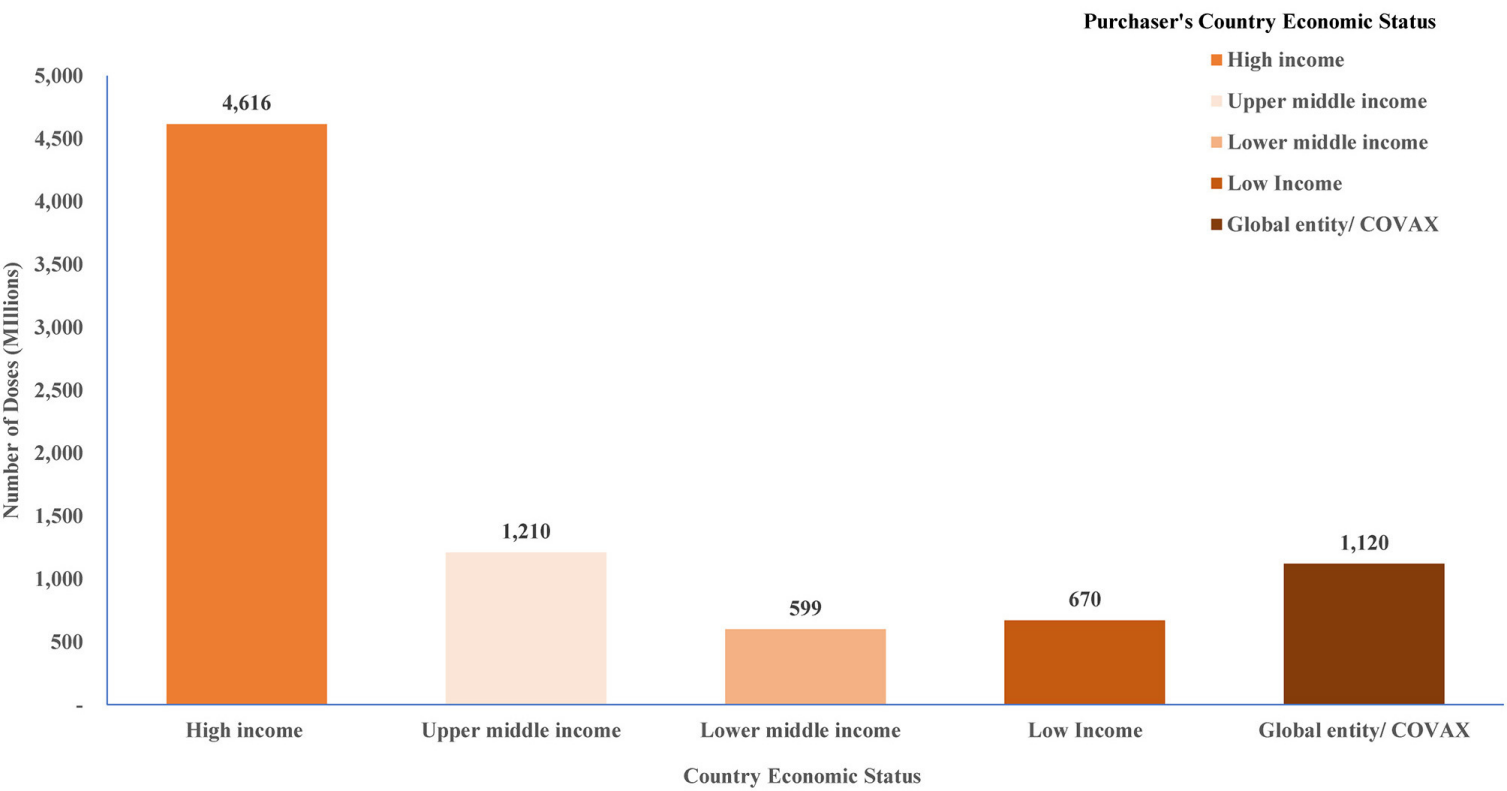
Confirmed number of doses purchased by country income level classification. Source: Duke Global Innovation Center (21).

This refers to a situation where the countries prioritize their own vaccine needs and push to get first access to the vaccines' supply during a transnational public health crisis (19). The world's richest countries procured and reserved more doses of the best COVID-19 vaccines to immunize their own populations multiple times and provide two or more booster shots (20). Figure 2 shows confirmed number of doses purchased by countries disaggregated by economic status. Among the HICs, by mid-August 2020, the USA had secured 800 million doses of at least 6 vaccines, the UK had purchased 340 million doses, followed by EU countries, Canada and Japan that had each ordered hundreds of millions of doses through advance market commitments to cover more than their entire populations (21, 22). By early 2022, HICs had stockpiled more doses of COVID-19 vaccine to provide their citizens with booster shots when some LICs had not fully administered a single dose to some of their populations (23). For LICs, purchases commenced in January 2021 through the African Union's pooled procurement approach leaving out many LICs in Latin America, Africa and Asia with few vaccines to cover their entire populations (22). This lowered the chances for LICs to access the vaccine on time as they were relatively out of stock.

#### 3.2.4. Weak health systems

The weak health infrastructures like limited laboratory facilities coupled with COVID-19 vaccine supply chain and logistics challenges contributed to vaccine inequity across LICs. Many health facilities in LICs lack proper cold storage facility with refrigerating and freezing temperatures to store vaccines at the recommended temperature like for Oxford-AstraZeneca at 2–8°C and the Pfizer at −70°C (5) which increases vaccines stock outs in very remote areas. To a greater extent, the geographical landscape of many LICs poses a significant challenge to distribute vaccines (5). Therefore, vaccines are delayed and sometimes arrive few months before their expiry date leaving little time for usage. Some countries in LICs are characterized by political instability and this results in untimely and inequitable distribution of the COVID-19 vaccine.

#### 3.2.5. Vaccine misconception

COVID-19 vaccine misconceptions remain a significant issue with regards equity. Recent studies investigated COVID-19 vaccine hesitancy, misconceptions and uptake in both LICs and HICs (24, 26). The existing literature cites the rapid pace of vaccine development as a primary reason for hesitancy in higher-income settings (24). Whilst reasons for hesitancy in LICs varied with most common ones being fear of adverse effects after vaccination, vaccination disinformation and myths or beliefs, religion, and lack of trust in the government and pharmaceutical companies (24, 25). For example, in Malawi, it was observed that many people have misconceptions about the COVID-19 vaccine, and believe it contains a microchip or deoxyribonucleic acid (DNA) marker. This conforms to the findings by World Health Organization (WHO) that despite increased supply of the vaccine through COVAX facility in Africa, only 28% of the adult population was vaccinated as of December 2022 (1). Low vaccine uptake is more prevalent in rural areas where myths and misconceptions about COVID-19 are most prevalent but are also areas that have less been hit by the pandemic. Thus, provision of COVID-19 vaccine alone, is only the first step to achieving the vaccine coverage required to tackle the pandemic.

### 3.3. The impact of vaccine inequity

COVID-19 vaccine iniquity continues to negatively impact progress on control of the pandemic. Firstly, the inequity resulted in low vaccination coverage in LICs. As the world is moving toward the global 70% vaccination coverage, the challenge remains as huge swathes of the population are being left behind especially in the LICs. For example, by December 2022, 22% of people in LICs were fully vaccinated compared to 75% in HICs (1).

Secondly, the opportunity for LICs to access COVID-19 supplies or vaccines on time decreased as they would be relatively out of stock consequently prolonging illnesses and deaths in LICs as only a small number of countries would get most of the supply on time. Illnesses and deaths among people reduced the availability of human resources needed to manage both COVID-19 and non-COVID-19 health burdens. Globally, its impact was projected at more than $1.2 trillion per year (27). It was estimated that in absolute terms, the USA incurred the largest relative cost of $15.7 billion in 2020–2021, expected to rise to $49.3 billion in 2023–2024 followed by Germany, at nearly $10 billion in 2020–2021 (27). Estimates also suggested that developing countries could lose at least US$220 billion in income, translating into higher debt and vulnerable growth in some of the world's poorest and most vulnerable countries (28). As governments across the globe would want to deal with this wreckage of their economies, it was estimated that 95 million more people would be pushed into extreme poverty, with another 200 million predicted to be at risk between the year 2020 and 2030 (7). This continued vaccine inequity is economically and epidemiologically damaging, leading to both long-lasting direct and indirect consequences.

Lastly, inequitable distribution allowed the SARS-CoV-2 to continue spreading, increasing the chances of resistance and emergence of new variants rendering already developed vaccines less effective (29). In an area of multiple variants with increased transmissibility, the demand for the COVID-19 services remained very high, stretching already overstretched health system. A study that evaluated the impact of COVID-19 on surgical services in Malawi revealed that when COVID-19 cases overwhelmed central hospitals, the provision of some other surgical services were compromised such that non-urgent or non-cancer surgical procedures were canceled or postponed in almost all of Malawi's central hospitals (30). An overwhelmed healthcare system compromises the management of urgent and emergent cases.

### 3.4. Potential solutions

To guide fair distribution, access and utilization of COVID-19 vaccines across LICs, proposed solutions from the discussions included.

#### 3.4.1. Expanding the COVAX facility

The COVAX facility- led by Gavi, the Vaccine Alliance, WHO and Coalition for Epidemic Preparedness Innovations (CEPI) was established solely to bring nations together, regardless of their income level, to ensure the procurement and equitable distribution of COVID-19 vaccines (31). More than a year since the COVAX facility initiated the delivery of the COVID-19 vaccines to LICs, development partners like the United Nations Children Fund (UNICEF) and manufacturers partnered to procure and deliver COVID-19 vaccine doses for almost 92 LICs and supported procurement for more than 97 upper middle-income and HICs (32). Ethically, by accepting the COVAX COVID-19 facility, all countries have a moral obligation not to leave behind or out the rest of the population unvaccinated. After all, we live in a global village and COVID-19 has been highly transmissible among others due to people's travel. Nonetheless, the COVAX initiative proved to be insufficient on its own as it covers only 20% of COVID-19 vaccine needs for all countries, far below the target population required for a coverage of 60% (33). This left an estimated financing gap of LICs, many of which are in Africa, of over $10 billion and $108 million in Sub-Saharan Africa (SSA) and Malawi, respectively (33). Either expanding the COVAX facility or taking advantage of other sources of funding like the World Bank COVID-19 facility and the African Export-Import Bank (Afrexim Bank) COVID-19 vaccine facility, among others, will increase allocation of COVID-19 vaccine in LICs.

#### 3.4.2. Waiving the control of intellectual property rights

Initially, as indicated above, India and South Africa proposed manufacturers in HICs relinquish intellectual property rights associated with COVID-19 vaccines, diagnostics and therapeutics to increase manufacturing and access in LMICs (17). Notwithstanding its importance, the Agreement on Trade-Related Aspects of Intellectual Property Rights (TRIPS agreement) waiver faced strong opposition from some HICs which host large domestic pharmaceutic industries e.g., European Union and UK, mainly on economic basis (34). The estimates suggested that BioNTech, the biotechnology company behind Pfizer's mRNA vaccine, alone could boost Germany's gross domestic product (GDP) by 0.5% in 2021 (35). Nonetheless, in 2022, the USA offered support to the TRIPS waiver with the key limitation that the waiver be issued to the production of vaccines only, not therapeutics and diagnostics (36). To achieve global vaccine equity, a nationalistic approach based on the welfare of the global community must be prioritized over private profits. Moreover, the waiver should go beyond vaccines to cover diagnostics and therapeutics as they are equally important to combat the SARS-CoV-2.

#### 3.4.3. Improving manufacturing capacity in LICs

As highlighted above, following the partial approval to the TRIPS waiver by the USA, manufacturing sites around the world were identified as candidates in scaling up vaccine production such that few countries like India, the Republic of Korea, Brazil, Indonesia, South Africa, Egypt, Morocco, Senegal, and Tunisia already started producing vaccines. Through the Africa Center for Disease Control (CDC), sites for vaccine clinical trials were established across the African continent (37). Currently, five COVID-19 vaccine clinical trials [e.g., Biological: Bacille Calmette-Guerin (BCG); SARS-CoV-2 rS/Matrix-M1 Adjuvant; Biological: ChAdOx1 nCoV-19 with placebo comparators] were established in South Africa. Other countries (e.g., Egypt, Tunisia, Senegal, Morocco, Ghana, Guinea Bissau, and Kenya) with varying capacities initiated trial activities in an attempt to facilitate successful vaccine implementation. India alone has the pre-existing infrastructure in place to manufacture more than three billion vaccines doses a year and a long-standing track record of cost-effective biosimilar vaccine manufacture on a large scale and a commitment to support equitable vaccine distribution and provide vaccine assistance to LICs (38). Nonetheless, patent holders rarely voluntarily agree to share manufacturing knowhow externally to facilitate this. Thus, IP flexibilities alone cannot address the shortage of infrastructure, capacity, technical knowledge, and regulatory recognition (39). Therefore, making vaccines available requires more collaboration among partners, increasing manufacturing capacity in LICs and providing new legal mechanisms to share technical production processes.

#### 3.4.4. Strengthen and improve health systems

Most LICs have weak health systems characterized by weak infrastructure such as limited laboratory facilities, and lack of cold storage facilities coupled with challenges of long distances to static vaccination sites. This consequently affects both the availability and uptake of vaccines. Investments should be made into strengthening the health systems as this would not only benefit the COVID-19 response, but also promote future pandemic preparedness, potentially creating a sustained impact for COVID-19 and other related pandemic's responses.

#### 3.4.5. Mass COVID-19 vaccination campaigns

Access to vaccines is one step toward vaccine equity but the negative public perceptions of the vaccine in LICs stall the progress. All the ongoing initiatives would be futile if populations across LICs remain hesitant to accept the vaccines. Studies on misconceptions and misperceptions of COVID-19 vaccine revealed that some misleading myths and numerous faith-related rumors led to the spread of wrong information on the cure of COVID-19 causing fears and mistrust of health care systems across affected countries (24, 25). To boost the COVID-19 vaccine uptake in LICs, the WHO, UNICEF, Gavi, the Vaccine Alliance, and partners are supporting mass vaccination drives in at least 10 priority countries to reach 100 million people by the end of April 2022 (9). This drive ignited a positive momentum against the pandemic, however, limiting the initiative to few populous countries will likely hinder the progress. Scaling up mass vaccination campaigns across all LICs where local communities have access to information, counseling, support related to COVID-19 vaccine will improve uptake. Moreover, designing an equitable and effective vaccine delivery plan for the populace will be equally important.

## 4. Conclusion

There are huge disparities in the coverage of COVID-19 vaccine in LICs. The key barriers to universal COVID-19 vaccine coverage are vaccine nationalism, low funding, low vaccine production on the African continent, high vaccine hesitancy, social media misinformation and misreporting, and lack of global health emergency policy on equitable access in a global pandemic. Currently, efforts through COVAX are recommendable in that they are promoting global solidarity by pulling together global COVID-19 resources to ensure equitable procurement, distribution, and COVID-19 vaccine access for all nations regardless of their income level. Therefore, the approach to COVID-19 vaccination needs to address key ethical and social justice concerns. Without global vaccine equity there cannot be global health security.

## Data availability statement

Publicly available datasets were analyzed in this study. This data can be found at: https://ourworldindata.org/covidvaccinations and https://public.tableau.com/app/profile/duke.global.health.innovation.center.

## Author contributions

JM-B and CK conceptualized, designed, and drafted the manuscript. JM-B, GC, WN, LN, SM, BC, DN, MC, CM, and AC critically reviewed the manuscript. All authors approved the final manuscript.
